# A global live cell barcoding approach for multiplexed mass cytometry profiling of mouse tumors

**DOI:** 10.1172/jci.insight.143283

**Published:** 2021-04-08

**Authors:** Soren Charmsaz, Nicole Gross, Elizabeth Jaffee, Won Jin Ho

**Affiliations:** Sidney Kimmel Comprehensive Cancer Center, Johns Hopkins University School of Medicine, Baltimore, Maryland, USA.

**Keywords:** Immunology, Oncology, Cancer, Cellular immune response, Mouse models

## Abstract

With the advent of cancer immunology, mass cytometry has been increasingly employed to characterize the responses to cancer therapies and the tumor microenvironment (TME). One of its most notable applications is efficient multiplexing of samples into batches by dedicating a number of metal isotope channels to barcodes, enabling robust data acquisition and analysis. Barcoding is most effective when markers are present in all cells of interest. While CD45 has been shown to be a reliable marker for barcoding all immune cells in a given sample, a strategy to reliably barcode mouse cancer cells has not been demonstrated. To this end, we identified CD29 and CD98 as markers widely expressed by commonly used mouse cancer cell lines. We conjugated anti-CD29 and anti-CD98 antibodies to cadmium or indium metals and validated their utility in 10-plex barcoding of live cells. Finally, we established a potentially novel barcoding system incorporating the combination of CD29, CD98, and CD45 to multiplex 10 tumors from s.c. MC38 and KPC tumor models, while successfully recapitulating the known contrast in the PD1-PDL1 axis between the 2 models. The ability to barcode tumor cells along with immune cells empowers the interrogation of the tumor-immune interactions in mouse TME studies.

## Graphical abstract

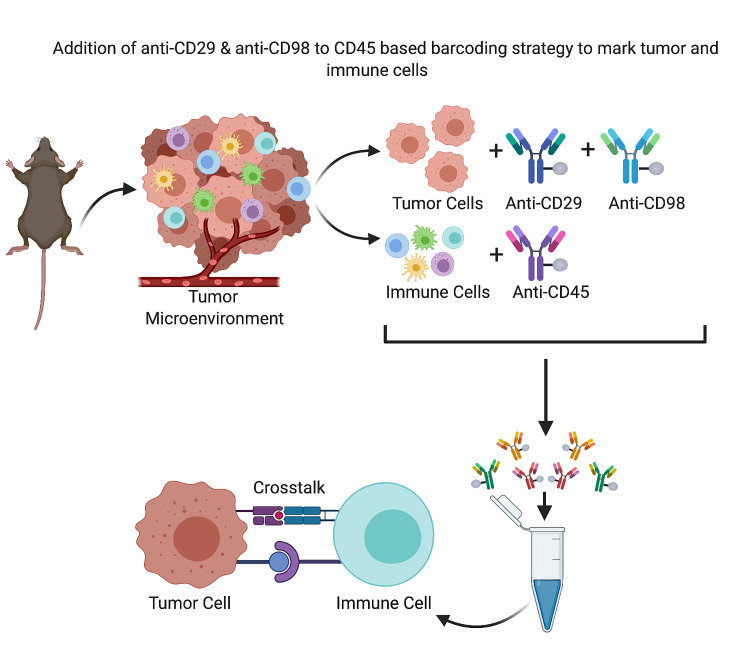

## Introduction

With remarkable progress in cancer immunology, mass cytometry or cytometry by TOF (CyTOF) have increasingly contributed to the understanding of the tumor microenvironment (TME) (1, 2). CyTOF is a technique that allows multiparametric analysis of single cells using metal tagged antibodies (3). Although similar to flow cytometry, CyTOF employs the use of antibodies conjugated to heavy metal ions rather than fluorophores. This strategy overcomes problematic spectra overlap inherent to fluorophores, allowing the parametrization of 40 or more markers per cell (4). CyTOF also obviates technical vulnerabilities associated with tandem fluorophores and issues related to autofluorescence, which often complicate the flow cytometry applications in studying epithelial/cancer cells, myeloid cells, abundance of debris, or intracellular markers (4). Importantly, due to the wider range of channels available for assaying, additional channels may be assigned to barcoding to allow multiplexing of samples for batched data acquisition. Barcoding improves throughput of data acquisition, reduces sample-to-sample variations, and provides a much more robust downstream analysis (5).

To barcode each sample effectively, it is critical to select markers that are present in all of the cells of interest. The first demonstration of multiplexed CyTOF by barcoding utilized Maleimido-mono-amide-DOTA (m-DOTA) (5). m-DOTA is a molecule that allows for the chelation of lanthanide metals to cellular thiol groups, and it is taken up by the cells (5). Naturally, this method requires cell permeabilization prior to barcoding, which was performed with methanol. Since some epitopes are sensitive to alcohol (6), permeabilization with methanol requires surface staining prior to barcoding. Alternatively, barcoding with transient partial permeabilization using saponin was demonstrated, allowing barcoding to be performed prior to surface staining (6). However, methods that depend on cell permeabilization also necessitate preceding cell fixation with paraformaldehyde. Fixation methods also disrupt the assayability of some markers, which limit barcoding applications. Thus, more recently, “live cell” barcoding strategies, in which surface markers are utilized, have been employed. Given the prevalent application of CyTOF methods in understanding immunological processes, a leukocyte common antigen CD45 was shown to be a reliable barcoding antigen to mark all live immune cells in a given sample (7, 8). However, to obtain an expanded view of the TME (and for any other applications that do not focus on immune cells), it is important to be able to also barcode nonimmune cells. To this end, it was found that β-2-microglobulin (B2M) and CD298, which mark MHC class I molecules and a subunit of sodium-potassium ATPase, respectively, are suitable for barcoding live human cells (9). While B2M and CD298 are useful for human samples, there are no validated markers that are robustly expressed by commonly used mouse cancer cell lines (e.g., MC38, CT26, B16, KPC, and Panc02), which are critical components of many syngeneic immunocompetent studies. Furthermore, there are presently no reliable antibodies for mouse CD298. Herein, to address this problem, we present a live cell barcoding approach for the multiplexing of mouse cancer cell lines and singly dissociated cells from mouse tumors.

## Results

### CD29 and CD98 identified as robust markers for labeling mouse cancer cell lines.

**Figure 1:**
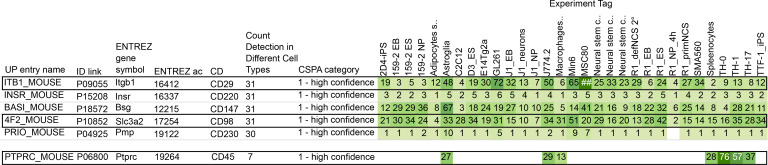
Potential surface markers for live barcoding of mouse cancer cell lines. The Cell Surface Protein Atlas utilizes Cell Surface Capture (CSC) technology to generate the cellular surfaceome snapshot. The database shows protein expression in various cell types and has filters that allow results to be narrowed. The following filters led to the identification of CD29 and CD98 as top hits in mouse cell lines: mouse, high confidence level, and CD markers, sorted in the descending order by the number of cell types in which the antigen was detected. The results for CD45 are shown for comparison. The numbers listed under each cell line indicate each CD marker’s unique peptide count in that cell type, while the color intensity increases with the number of unique peptide counts.

**Figure 2:**
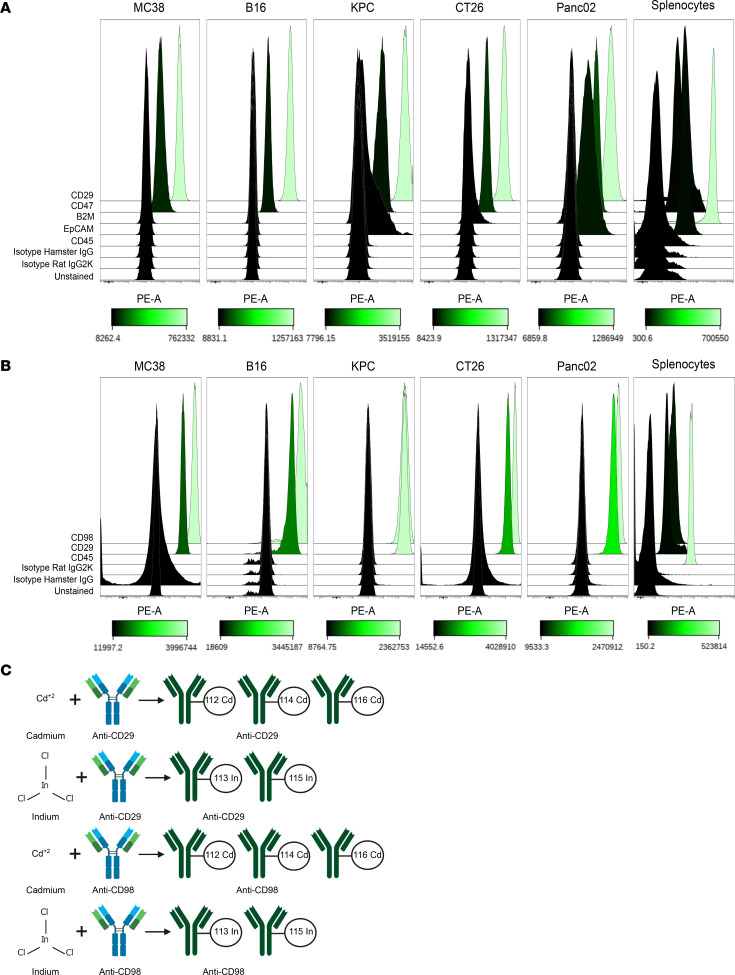
Analysis of candidate pan-tumor markers in various cancer cell lines. (**A**) Flow cytometry was utilized to test the expression of the following candidate pan-tumor markers: CD29, CD47, B2M, and EPCAM, in addition to CD45, isotype hamster IgG, and isotype rat IgG2K, which served as controls. (**B**) CD98 expression was tested separately via flow cytometry in addition to CD29, CD45, isotype hamster IgG, and isotype rat IgG2K, which served as controls. These markers were tested in several cancer cell lines: MC38, B16, KPC, CT26, and Panc02, in addition to splenocytes. Splenocytes were harvested from female BALB/cJ. CD29 and CD98 were identified as the most robustly expressed markers across cancer cell lines. (**C**) Anti-CD29 and anti-CD98 antibodies were covalently conjugated to the following isotopes: cadmium (112, 114, and 116 Cd) and indium (113 and 115 In).

In order to barcode all cells originating from tumors, in addition to immune cells, we needed to identify markers that are widely expressed on the surface of the cancer cell lines. To find potential candidate markers, we utilized the Cell Surface Protein Atlas, which is an interactive database developed using Cell Surface Capture (CSC) technology and mass spectrometry to generate cellular surfaceome snapshots (10) (Supplemental Figure 1; supplemental material available online with this article; https://doi.org/10.1172/jci.insight.143283DS1). Using this resource, we found CD29 and CD98 as potential candidates, since they are detectable in all 31 different mouse cell lines (which includes immune cells, embryonal stem cells, glial cells, adipocytes, and myocardial cells) within the database (Figure 1 and Supplemental Table 1). As a point of reference, we also examined the results for CD45, and as expected, CD45 was detectable in only 7 of the 31 different mouse cell lines within the database (Figure 1). CD29 is a surface marker involved in cell adhesion (11), while CD98 is a transmembrane protein involved in amino acid transport (12), and both are highly expressed also in mouse CD45^–^ stromal cell types (ImmGen, Supplemental Table 2). CD29 and CD98 were particularly feasible markers to be tested, given the commercial availability of purified monoclonal anti-mouse antibodies. Other notable top hits included CD298, CD54, and CD47 (Supplemental Table 1). In addition to CD29 and CD98, we also decided to test the following antigens, due to their known functions that make them candidates to be widely expressed on the cancer cell lines: CD47, B2M, and Epithelial cell adhesion molecule (EPCAM). CD47 is a cell surface receptor that regulates macrophages from attacking healthy cells and is known to be overexpressed in cancers (13); B2M is a marker on MHC class 1 molecules (9); and EPCAM is a transmembrane receptor on epithelial cells that aids cell adhesion (14). CD45, isotype hamster IgG, and isotype rat IgG2K were also tested as controls. In order to validate the utility of these candidates as barcoding markers, we tested their expression in the following commonly used cancer cell lines: MC38 (colon carcinoma) (15, 16), CT26 (colon carcinoma) (16, 17), B16 (melanoma) (16, 18, 19), Panc02 (pancreatic ductal adenocarcinoma) (16, 20), and KPC (pancreatic ductal adenocarcinoma) (21) (Supplemental Table 3). These antigens were also tested on splenocyte samples to examine their relative expression in immune cells. CD29 and CD98 were tested separately, with CD29 first being compared against the aforementioned candidate markers (Figure 2A) and CD98 subsequently being tested in reference to CD29 (Figure 2B). Of all of the antigens tested, CD29 and CD98 were the most broadly expressed antigens among cancer cell lines, with CD98 having slightly higher expression than CD29 in MC38, B16, CT26, and Panc02 but almost equal expression in KPC (Figure 2, A and B). CD47 showed the next highest expression among cancer cell lines, while the remaining antigens had relatively similar levels of expression, with the exception of EPCAM having a considerable level of expression in Panc02 (Figure 2A). CD29 and CD98 expression was reliably present in splenocytes but was less intense compared with the expression of CD45, demonstrating that CD29 and CD98 may not be as robust as CD45 for barcoding immune cells (Figure 2, A and B). To note, all cancer cell lines had much higher levels of autofluorescence compared with splenocytes. After finding that CD29 and CD98 are highly expressed in the tested mouse cancer cell lines, anti-CD29 and anti-CD98 antibodies were conjugated to 3 different cadmium isotopes (112Cd, 114Cd, and 116Cd) and 2 different indium isotopes (113In and 115In) (Figure 2C). Finally, 116Cd-CD29 and 112Cd-CD98, in addition to 114Cd-CD45 as a control, were utilized to confirm that these antigens are robustly assayable in our cell lines via CyTOF (Supplemental Figure 2).

### Validation of CD29- and CD98-based barcoding systems.

**Figure 3:**
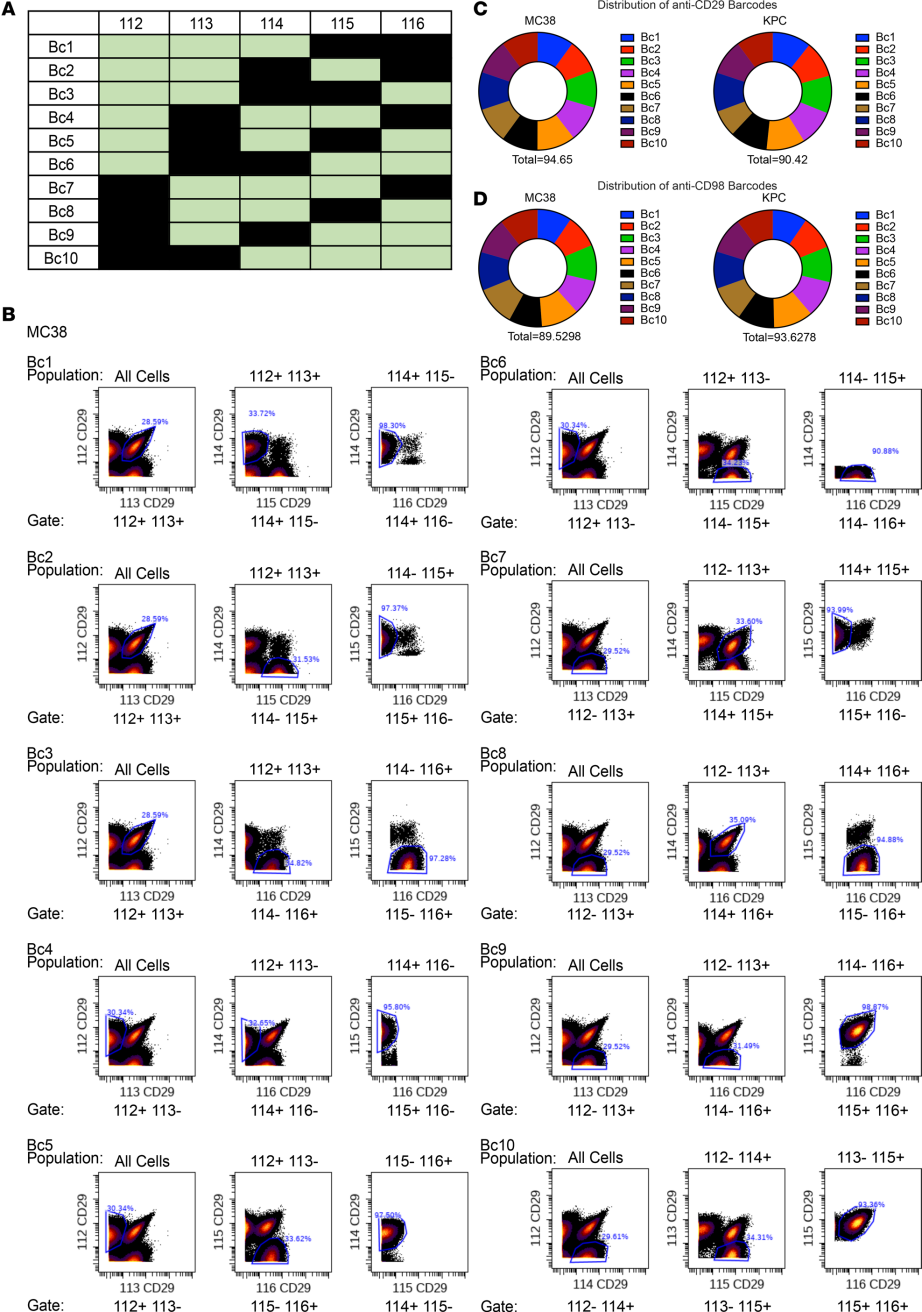
Testing optimized CD29 barcoding system. (**A**) Using 112Cd-, 113In-, 114Cd-, 115In-, and 116Cd-conjugated anti-CD29 and anti-CD98 antibodies, 10 unique barcodes were established for both anti-CD29 and anti-CD98 utilizing a 5-choose-3 barcoding strategy. MC38 and KPC cancer cell lines were stained using the aforementioned antibodies at a concentration of 0.25 μg/100 μL. (**B**) Ten-plexed MC38 and 10-plexed KPC samples were debarcoded using their respective gating hierarchies back to their original individual samples. Representative gating schema for the MC38 batch stained with anti-CD29 barcodes are shown. (**C** and **D**) Distribution of individual anti-CD29 barcodes (**C**) and anti-CD98 barcodes (**D**) yielded from the debarcoding process of MC38 and KPC samples. Both CD29 and CD98 barcoding systems resulted in a fairly even distribution of all 10 barcodes in MC38 and KPC samples.

Having conjugated anti-CD29 and anti-CD98 antibodies and validated their stainability with CyTOF, we next tested the potentially novel barcoding methods using the previously described 112Cd-, 113In-, 114Cd-, 115In-, and 116Cd-conjugated anti-CD29 and anti-CD98 antibodies. We employed the use of a 5-choose-3 barcoding strategy to yield 10 unique barcodes for both CD29 and CD98 (Figure 3A). Ten wells of MC38 and 10 wells of KPC cancer cell lines were stained with both anti-CD29 and anti-CD98 separately at a concentration of 0.25 μg/100 μL based on testing at several dilutions (Supplemental Figure 3). After each well was stained with a unique barcode, MC38 and KPC cells were multiplexed into four 10-plex batches, 1 for MC38 with anti-CD29 barcodes, 1 for KPC with anti-CD29 barcodes, 1 for MC38 with anti-CD98 barcodes, and 1 for KPC with anti-CD98 barcodes. Upon data acquisition, the resulting cell events were manually debarcoded by hierarchal gating for cells that are positive for the 3 barcode channels and negative for the remaining 2 barcodes. This strategy was used for both anti-CD29 and anti-CD98 barcodes (see representative results for MC38 and for KPC in Figure 3B and Supplemental Figure 4, respectively). Event counts revealed that the anti-CD29 barcoding strategy successfully captured 94.65% of all MC38 cells and 90.42% of all KPC cells, while the anti-CD98 barcoding strategy successfully captured 89.53% of all MC38 cells and 93.63% of all KPC cells. Furthermore, both anti-CD29 and anti-CD98 barcodes accounted for roughly 10% of the stained cells for both MC38 and KPC, demonstrating effective 10-plex runs based on CD29 and CD98 (Figure 3, C and D). We also employed a previously published algorithm (22) for debarcoding of the samples to see whether the barcoding strategy is robust to automation. Applying a separation threshold of 0.3, 77% and 84% of MC38 and KPC cells, respectively, could be reliably assigned to each barcode using anti-CD29, and 76% and 86% of MC38 and KPC cells, respectively, could be reliably assigned to each barcode using anti-CD98 (Supplemental Figure 5).

### Live cell barcoding system allows multiplexed mouse tumor profiling and recapitulates the presence of PD1-PDL1 axis in MC38 tumors.

**Figure 4:**
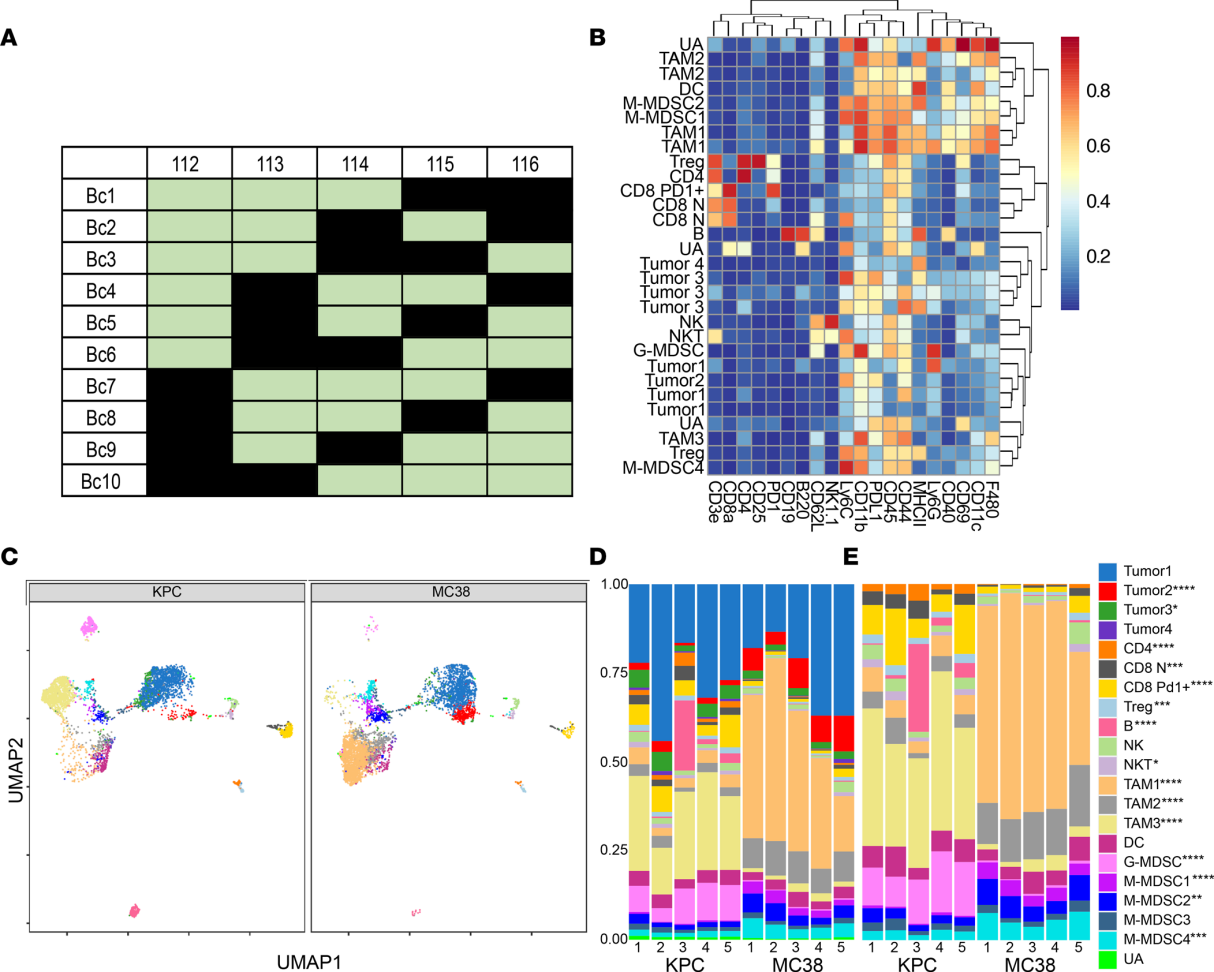
Demonstration of the barcoding strategy on mouse tumors. (**A**) Using 112Cd-, 113In-, 114Cd-, 115In-, and 116Cd-conjugated anti-CD45, anti-CD29, and anti-CD98 antibodies, 10 unique barcodes were established based on a 5-choose-3 barcoding strategy. (**B**) Based on a data set using 20 markers in a mouse immune profile mass cytometry panel, the FlowSOM algorithm was then used to generate 30 metaclusters annotated into 21 final clusters. Displayed is the expression heatmap for all of the samples in the data set. (**C**) Phenotype clusters identified by FlowSOM clustering shown as a UMAP plot. (**D**) Stacked bars represent the percentage of total live cells per mouse tumor within each cluster. (**E**) Stacked bars represent the percentage of CD45^+^ cells for each mouse tumor within each live cell cluster. Color legends on the right apply to **B**–**D**. Cell type abundances with significant difference between the KPC and MC38 are annotated. FDR-adjusted *P* values by edgeR: ****P < 0.0001, ***P < 0.005, **P < 0.01, *P < 0.05. CD8 N, CD8^+^ naive T cells; TAM, tumor-associated macrophages; G-MDSC/M-MDSC, granulocytic/monocytic myeloid-derived suppressor cells; UA, unassigned.

**Figure 5:**
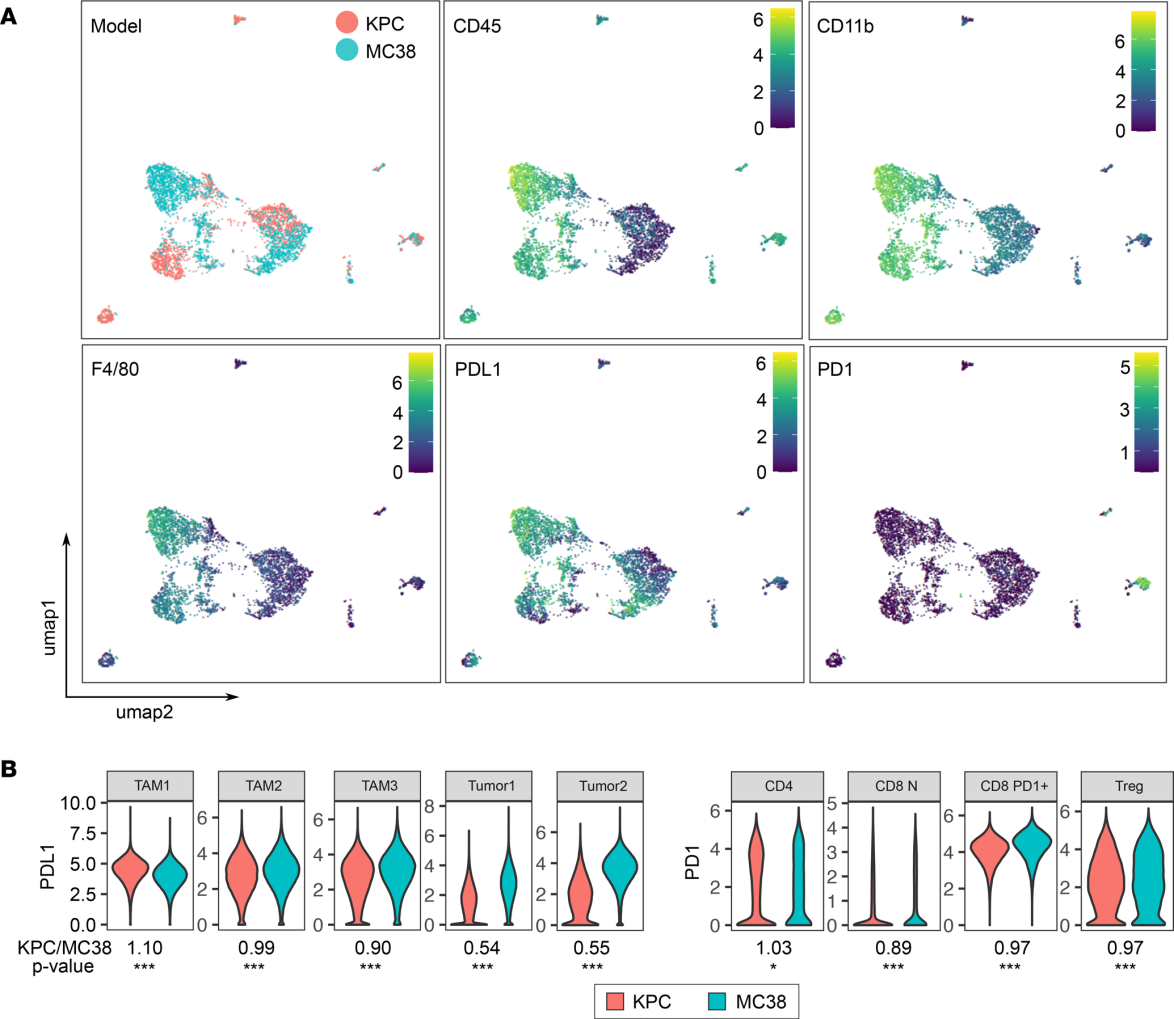
Comparison of PDL1-PD1 axis between KPC and MC38 tumor models. (**A**) Representative UMAPs for the tumor model, CD45, CD11b, F4/80, PDL1, and PD1 expression. (**B**) Violin plots comparing the expression levels of PDL1 and PD1 in specific cell type clusters with annotations of median fold difference (KPC/MC38) and FDR-adjusted *P* values by edgeR: ***P < 0.005, *P < 0.05. Number of cells for each comparison of KPC versus MC38: TAM1, 55,607 versus 519,369; TAM2, 45,595 versus 122,957; TAM3, 373,020 versus 26,437; Tumor1, 511,692 versus 396,897; Tumor2, 32,692 versus 107,499; CD4, 30,656 versus 3229; CD8 N, 38,463 versus 6,339; CD8 PD1^+^, 102,213 versus 17,319; Treg, 23,766 versus 7928. CD8 N, naive CD8+ T cells; TAM, tumor-associated macrophages.

Finally, to demonstrate the use of this barcoding system on mouse tumors, we used a s.c. inoculated syngeneic immunocompetent mouse model using KPC and MC38 cells. Tumors were harvested and enzymatically dissociated into single cells. To note, single cell dissociation of s.c. tumors involves Percoll separation of the enzymatically dissociated samples to enrich for immune cells by filtering out debris, dead cells, and RBCs. We multiplexed 10 single cell dissociated tumor samples (5 KPC samples and 5 MC38 samples) using the combination of anti-CD29, anti-CD98, and anti-CD45 markers on the same channels to barcode based on the same 5-choose-3 scheme for each barcoding marker (Figure 4A). We performed CyTOF using a panel of 20 markers to profile the tumors (Supplemental Table 4), and a clustering analysis of the resulting CyTOF data using the FlowSOM algorithm (see Methods) identified 30 metaclusters that could be annotated into 21 final clusters (Figure 4, B and C, and Supplemental Table 5). Using our barcoding strategy, we could observe several differences in the TME between MC38 and KPC tumors, both as a percentage of live cells and as a percentage of CD45 cells, enabling analysis separately for the nonimmune and immune compartments. Within the nonimmune compartment, the CD45^–^ or CD45^dim^ cells, the vast majority of which are presumably cancer cells, belonged to 4 major phenotypically distinct clusters. The most abundant CD45^–^ cell cluster was substantially represented in both KPC and MC38 tumors, while the second most abundant CD45^–^ cell cluster was primarily observed in MC38 tumors (Figure 4D). Within the immune compartment, KPC tumors had significantly more infiltrating lymphoid cells (B cells, T cells, NK cells), and MC38 tumors in general had more tumor-associated macrophages (TAM) — but also specifically TAM clusters 1 and 2. MC38 tumors also exhibited higher percentages of monocytic myeloid-derived suppressor cells (M-MDSCs), whereas KPC tumors had greater abundance of granulocytic MDSC (G-MDSCs) (Figure 4, D and E, and Supplemental Figure 6). When we examined in detail the functional phenotypes of these clusters that were differentially abundant, we found MC38 tumors exhibited striking predominance of PDL1-expressing clusters in both immune and nonimmune compartments (Tumor 2 [CD45^–^ cell cluster], TAM1, TAM2, M-MDSC1, M-MDSC2) (Figure 4 and Figure 5A). While there was an abundance of PD1^+^CD8^+^ T cells in the KPC tumors (Figure 4, D and E), abundance of PDL1-expressing cells were markedly lower (Figure 5A), suggesting that the immune suppression within the s.c. KPC tumors may not be heavily dependent on the PD1-PDL1 axis. Furthermore, when we compared the expression levels of PDL1 at the per-cell level within each cell type cluster (i.e., cell states instead of cell type abundances), the top 2 CD45^–^ cell clusters, annotated as Tumor 1 and Tumor 2, showed substantial and statistically significant differences in PDL1 expression, again showing that MC38 tumors have higher PDL1 expression within the CD45^–^ compartment (Figure 5B). These results are consistent with previous observations including our own showing that s.c. MC38 tumors respond well to anti-PD1 therapy in both early- and late-treatment paradigms, whereas s.c. KPC tumors do not (23–26). In particular, our analysis of the PD1-PDL1 axis by simultaneously profiling both the nonimmune and immune compartments further supports the previously established role of PDL1 expression in both the tumor cells and TAMs in the immune escape of MC38 tumors (25, 27–29).

## Discussion

In this study, we were able to establish both CD29 and CD98 as useful markers to barcode commonly used mouse cancer cell lines and demonstrate its utility in a 10-plex barcoding system in live mouse tumor samples. Whereas anti-CD45 alone only offers staining of immune cells, the combination of anti-CD29, anti-CD98, and anti-CD45 antibodies for barcoding is a potentially novel strategy that allows for the staining of mouse immune and tumor cells in any given sample. Moreover, the dual staining of tumor cells with both anti-CD29 and anti-CD98 intuitively provides additional robustness for multiple reasons: (a) the expression of either of the 2 markers may change depending on the biological context, (b) higher technical fidelity in the staining process, and (c) the existence of more than 1 option in cell lines that we did not test. We have also demonstrated the ability of the 5-choose-3 barcoding scheme to successfully debarcode multiplexed samples back into individual samples both in manual and automated methods. Our barcoding approach is especially valuable when profiling multiple different conditions and comparing across tumor models that are composed of varying nonimmune and immune components within the TME. It also provides the ability to look at cellular compositions as a percentage of total cells, which can be less predictable depending on the dissociation protocol used.

Importantly, our application of cadmium metals for barcoding, which has only been recently made available commercially, in conjunction with indium channels were particularly advantageous since — despite the relatively lower mass-signal characteristics of these channels (channels 112–116) — they were used to target highly abundant markers and do not interfere with the signal of the channels dedicated to assaying the markers of interest (140 or more) (9). Also, by increasing the number of total metals used for barcoding with the addition of another channel (e.g., 89Y or 194-198Pt), we could easily expand the current 10-plex scheme to 20-plex (6-choose-3) or theoretically more as needed. By increasing the number of samples multiplexed and leveraging the ability to multiplex samples upstream of any staining step without fixation or permeabilization for both immune and nonimmune/tumor cells conserves reagents, decreases risk of batch variability, and allows us to rely on having fewer cells per sample. Lastly, CD29 and CD98 may be used as a barcoding target for proteogenomics platforms (e.g., CITE-seq) and a membrane marker in emerging multiplexed imaging techniques to improve cell segmentation in mouse models. Although these applications will require further experimental validation, our study establishes the rationale.

Our study has the following limitations. First, the metal-conjugated antibodies were conjugated across the span of several days, leading to variations in staining quality for each antibody. This lot-to-lot variation is expected and requires testing each time. The second limitation is that CD29 and CD98 expression is not inherently tumor cell specific, and they may or may not be present in other nonimmune cells. Thus, further extension of the application by including other phenotyping markers to identify less common cell types present in the CD45^–^ compartment such as podoplanin (for stromal cells) and CD31 (endothelial cells) would be valuable. Also, though we have tested and shown that both CD29 and CD98 are robust in several cancer cell lines, barcoding cancer cell lines not tested in this study will require independent verification in those cell lines.

In summary, we have shown the utility of CD29 and CD98, in addition to CD45, as robust markers for barcoding in studies involving mouse tumors to permit interrogation of the TME beyond the immune compartment. This study expands the applicability of suspension mass cytometry and broadens the scope of inquiry into cancer-stroma interactions and therapy responses, especially in the context of targeting the TME.

## Methods

### Cell Surface Protein Atlas.

In order to identify CD29 and CD98 as pan-tumor markers, we utilized the Cell Surface Protein Atlas (10). First, we used the explore option for the downloadable “Matrix of all proteins and their detection in the different cell types” to open the relevant database. The cell types included in the database are primary cells from neural, myocardial, immune, pancreatic, and glial cells, among other types. From there, filters were used to select for the following: mouse, high confidence level, and CD markers. After sorting the results in descending order for “count of detection in different cell types,” the results in Figure 1 and Supplemental Table 1 could be generated.

### ImmGen.

To validate the CD29 and CD98 expression in nonimmune cell types using another searchable external database, we used ImmGen (30) to explore the RNA sequencing expression of *Itgb1* (CD29) in stromal mouse cells available in the database. Among the reference populations, 3 were identified as CD45^–^. Expression levels of *Igtb1* (CD29), *Slc3a2* (CD98), *Ptprc* (CD45), and *Pdpn* in all reference populations were tabulated separately in Supplemental Table 2.

### Antibodies.

The mass cytometry antibodies and isotopes that were used for the duration of the experiment are shown in Figure 2C. Conjugation of the listed antibodies was carried out using Maxpar conjugation/metal labeling kits. The process was performed following the manufacturer’s protocol associated with the respective kits. To begin, 50 Kda ultra filtration columns (Amicon) were used to perform a buffer exchange protocol on purified antibodies (BioLegend) that were then partially reduced with 4 mM TCEP (Thermo Fisher Scientific). Concurrently, polymers were loaded individually with isotopically enriched metals, 112Cd (Fluidgim), 113In (Trace Sciences), 114Cd (Fluidigm), 115In (MilliporeSigma), and 116Cd (Fluidigm). Metal-loaded polymers yielded from this process were then conjugated to their respective antibodies. All conjugated antibodies were then subjected through a series of washing steps using a wash buffer before their concentrations in a wash buffer were quantified using Nanodrop. Final antibody concentrates were then diluted in their respective stabilization buffer. MCP9 polymer-conjugated antibodies were diluted in HRP-Protector (Boca Scientific) while X8 polymer-conjugated antibodies were diluted in a stabilization buffer (Candor) containing 0.3% sodium azide. Each unique antibody was then tested against 4 serial dilutions (0.0625 μg/100 μL, 0.125 μg/100 μL, 0.25 μg/100 μL, and 0.5 μg/100 μL) to ensure sufficient signal upon staining.

### Cell culture.

All cell lines were thawed and in culture for more than a week, and 2 passages were conducted for each cell line before experimental use. MC38 Cells (Kerafast) were maintained in DMEM-based media (Thermo Fisher Scientific) containing 10% FBS (Gemini), 1% L-glutamine (Thermo Fisher Scientific), 1% penicillin/streptomycin (Thermo Fisher Scientific), 1% HEPES (Thermo Fisher Scientific), 1% sodium pyruvate (MilliporeSigma), and 1% nonessential amino acids (Thermo Fisher Scientific) in 5% CO_2_ at 37°C. B16-F10 (ATCC) cells were maintained in DMEM-based media containing 10% FBS, 1% penicillin/streptomycin, 1% sodium pyruvate, and 1% nonessential amino acids in 5% CO_2_ at 37°C. CT26 (ATCC) cells were maintained in RPMI 1640 with glutamine (Thermo Fisher Scientific) containing 10% FBS, 1% penicillin/streptomycin, 1% sodium pyruvate, and 1% nonessential amino acids in 5% CO_2_ at 37°C. KPC cells were derived from transgenic mice harboring pancreas-specific KrasG12D and p53R172H mutations (21) and were maintained in RPMI 1640 with glutamine containing 10% FBS, 1% penicillin/streptomycin, 1% sodium pyruvate, and 1% nonessential amino acids in 5% CO_2_ at 37°C. Panc02 cells established and authenticated as previously described (20, 31) were maintained in DMEM-based media containing 10% FBS, 1% penicillin/streptomycin, and 1% L-glutamine (Thermo Fisher Scientific) in 10% CO_2_ at 37°C.

### MC38 and KPC tumor model.

Mice were purchased from The Jackson Laboratory and allowed to acclimate to the facility environment for 1 week prior to experimental use. MC38 and KPC cells were then s.c. injected in a syngeneic immunocompetent C57BL/6J background. Cells were injected at a concentration of 5 × 10^5^ cells per injection volume of 100 μL into the right hind limb of 6-week-old female C57BL/6 mice and were left to grow for 15 days. On day 15, mice were euthanized, and tumors were harvested and placed in incomplete media on ice before being enzymatically dissociated. Tumors were transferred to gentleMACS C tubes (Miltenyi Biotec) to be enzymatically dissociated using a mouse Tumor Dissociation Kit (Miltenyi Biotec) enzymatic mix in combination with the use of a gentleMACS Octo Dissociator (Miltenyi Biotec), using the built-in heated mouse tumor protocol. The resulting homogenate was then transferred to 50 mL conical tubes containing complete RPMI media through 100 μm strainers in order to both filter and quench samples. Homogenates were then subject through another cleaning step that involved gradient centrifugation using Percoll (GE Healthcare). Homogenates were resuspended in 40% percoll and underlaid with 80%; they were then centrifuged to separate single cells from debris, dead cells, and RBCs. Resulting single cells were then finally washed with complete RPMI, and pellets of single cells were obtained.

### Flow cytometry.

Fluorescent flow cytometry on samples was conducted with the following 2 panels. Panel 1 consisted of: anti–CD29 PE (HMb1-1, BioLegend, 1:100), anti–CD47 PE (Miap301, BioLegend, 1:100), anti–Β2M PE (A16041A, BioLegend, 1:100), anti–EPCAM PE (G8.8, BioLegend, 1:100), anti–CD45 PE (30-F11, BioLegend, 1:100), anti–isotype hamster IgG PE (HTK888, BioLegend, 1:100) ,and anti–isotype rat IgG2a k PE (RTK2758, BioLegend, 1:100). Panel 2 consisted of: anti- CD98 (RL388, BioLegend, 1:100), anti–CD29 PE (HMb1-1, BioLegend, 1:100), anti–CD45 PE (30-F11, BioLegend, 1:100), anti–isotype rat IgG2a k PE (RTK2758, BioLegend, 1:100), and anti–isotype hamster IgG PE (HTK888, BioLegend, 1:100). Control splenocytes were harvested from a healthy 8-week-old Balb/cJ female mouse (The Jackson Laboratory). Cancer cells in flasks were trypsinized and counted using a Trypan blue dilution and a hemocytometer. One million cells per well were plated and washed. Following an initial wash, cells were then Fc blocked for 10 minutes at 4°C. Samples were then stained at a concentration of 0.2 μg/100 μL and shielded from light for 30 minutes at 4°C. Before data acquisition on a Beckman CytoFLEX, FACS buffer was used to wash the cells a total of 2 times. Supervised gating analysis was then performed on Cytobank.

### Mass cytometry staining and data acquisition.

Eight wells of MC38 cells, 8 wells of splenocytes, 10 wells of MC38 cells, and finally 10 wells of KPC cells were plated on a 96-well plate at 1 × 10^6^ cells per well. On a separate 96-well plate, 10 wells of MC38 and 10 wells of KPC were plated at 5 × 10^5^ cells per well. Initially, plated cells were washed with PBS and 2 mM EDTA. This was then followed by a 5-minute incubation in palladium chloride that was dissolved in DMSO (MilliporeSigma) and then diluted in PBS to a concentration of 500 nM. This incubation at room temperature is used to mark viability. Following the 5 minutes, RPMI containing 10% FBS was added to the cells in order to quench any residual palladium (9). All wells were then stained with a unique metal barcode within their sample type for 25 minutes at room temperature, followed by a series of washes with a cell staining buffer (Fluidigm). The 16 single samples (8 wells of MC38 and 8 wells of splenocytes) were then transferred into tubes via 40 μm filters, while both sets of 10 wells of MC38 cells and both sets of 10 wells of KPC cells were 10 plexed (batched) and then transferred into tubes via 40 μm filters. After a series of washes, the cells were then stored in 1% methanol-free formaldehyde diluted in PBS (Thermo Fisher Scientific) until the day of data collection.

Single cell dissociated samples from mouse MC38 and KPC tumors were plated into 10 respective wells on a 96-well plate at 1.5 × 10^6^ cells per well. Initially, plated cells were washed with PBS and 2 mM EDTA. This was then followed by viability staining as above. The 10 wells were then stained with a unique metal barcode to differentiate between samples for 25 minutes at room temperature, followed by a series of washes with a cell staining buffer (Fluidigm). The 5 samples were then batched and transferred into a 10-plexed tube using a 40 μm filter. The multiplexed tube was then blocked with 1 μg anti-mouse Fc block (BD Biosciences, 553142, clone 2.4G2) for 10 minutes at room temperature, followed by a surface staining cocktail of antibodies (Supplemental Table 4) performed in cell staining buffer for 30 minutes at room temperature. After a series of washes, the cells were then stored in 1% methanol-free formaldehyde diluted in PBS (Thermo Fisher Scientific) until the day of data collection.

On the day of data collection, before samples were run, samples were stained with rhodium (Fluidigm) diluted at 1:500 in Maxpar Fix and Perm Buffer (Fluidigm) for 45 minutes at room temperature. Samples were run, and events and mass cytometry data were acquired on a Helios mass cytometer (Fluidigm) at the University of Maryland School of Medicine Center for Innovative Biomedical Resources (CIBR) Flow Cytometry and Mass Cytometry Core Facility (Baltimore, Maryland, USA).

### Mass cytometry data processing and analysis.

All CyTOF analysis was performed as previously described (24, 32). Briefly, acquired data were randomized, and beads were normalized and then removed using CyTOF software (Fluidigm) v6.7. Gating based on Rh+ intensity (DNA-based cell ID) and the event length parameter were used to identify cell events, and whenever used, dead cells were gated out based on palladium intensity. Debarcoding of the preprocessed batches was manually performed by hierarchal gating using Cytobank (v7.3.0) or FlowJo (v10.6.1). Automated debarcoding was performed using the Single Cell Debarcoder (22) within the *premessa* package (https://github.com/ParkerICI/premessa/commit/467d64150297d83832c3960750ac4792c99153fd). Clustering analysis was performed using FlowSOM (33) algorithm and visualized by UMAP (34) in R (v3.6.2), based on a modified pipeline from a prior report by Nowicka et al. (35). The data set was clustered into 30 metaclusters that were annotated into major immune cell types based on canonical markers (e.g., CD45^+^CD3^–^CD19^+^B220^+^ representing B cells). Further subtyping of immune cell types was carried out based on key functional markers (e.g., PD-L1 and MHC-II). Some metaclusters shared common marker expression profiles and were merged into a final annotated cluster (Supplemental Table 5).

### Figure generation.

Figure 1 was screen captured from the user-interface Cell Surface Protein Atlas. Figure 2, A and B, and Figure 3B were acquired from Cytobank. Figure 2C was created on Biorender. Figure 3A was created in Microsoft Excel. Figure 3, C and D, was generated in GraphPad PRISM 8 (v8.3.1) using data originating from Cytobank. Figure 4A was created in Microsoft Excel. Figure 4, B–E, was generated in R. Any figure arrangement was done in Inkscape (v1.0 for Windows or v1.0.0rc1 for MacOS).

### Statistics.

To analyze differential abundances of the clusters, we used edgeR as previously published (35, 36) to fit models and perform moderated tests at the cluster level while sharing information on variance across all samples. Raw *P* values were adjusted by FDR.

### Study approval.

Experiments and euthanasia were performed in accordance with Johns Hopkins IACUC–approved protocols.

## Author contributions

SC and NG designed and performed experiments, analyzed data, wrote the manuscript, and revised the manuscript. EJ analyzed data, revised manuscript, and provided funding. WJH conceived the study, designed experiments, analyzed data, wrote the manuscript, revised the manuscript, and provided funding. Order of co–first authors was determined based alphabetical order of last names.

## Supplementary Material

Supplemental data

Supplemental Table 1

Supplemental Table 2

Supplemental Table 5

## Version 1 03/09/2021

In-Press Preview

## Version 2 04/08/2021

Electronic publication
