# Supplementary material for: A global live cell barcoding approach for multiplexed mass cytometry profiling of mouse tumors
Source: JCI Insight. 2021 Apr 8;6(7):e143283. doi: 10.1172/jci.insight.143283 (PMC8119183; doi:10.1172/jci.insight.143283)
Supplement: Supplemental data [file jciinsight-6-143283-s011.pdf]

SUPPLEMENTARY DATA

Supplementary Figures 1-6

Supplementary Table S1. Top candidate surface markers for barcoding from Cell Surface Protein Atlas (*separately attached as an excel file*)

Supplementary Table S2. Mouse RNAseq data from ImmGen (Reference Populations) (*separately attached as an excel file*)

Supplementary Table S3. Mouse cell line information

Supplementary Table S4. Mouse immune profiling panel

Supplementary Table S5. Cell type annotations (*separately attached as an excel file*)

References Related to Supplementary Table S3

Supplementary Figure 1. Cell Surface Protein Atlas

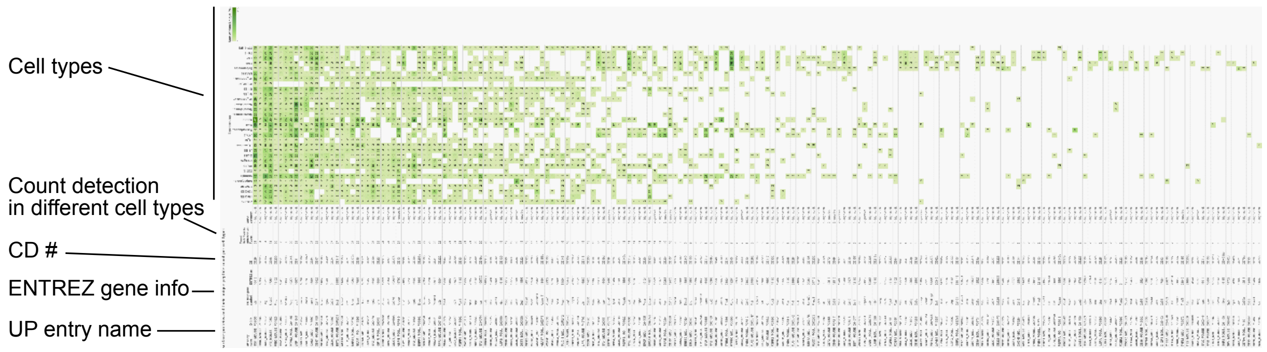

The Cell Surface Protein Atlas utilizes Cell Surface Capture (CSC) technology to generate the above cellular surfaceome snapshot. The database shows protein expression in various cell types and has filters that allow results to be narrowed. This representative snapshot was captured prior to applying filters and serves only to give an overview of the resource.

Supplementary Figure 2. CD29 and CD98 expression in mouse cancer cell lines via CYTOF

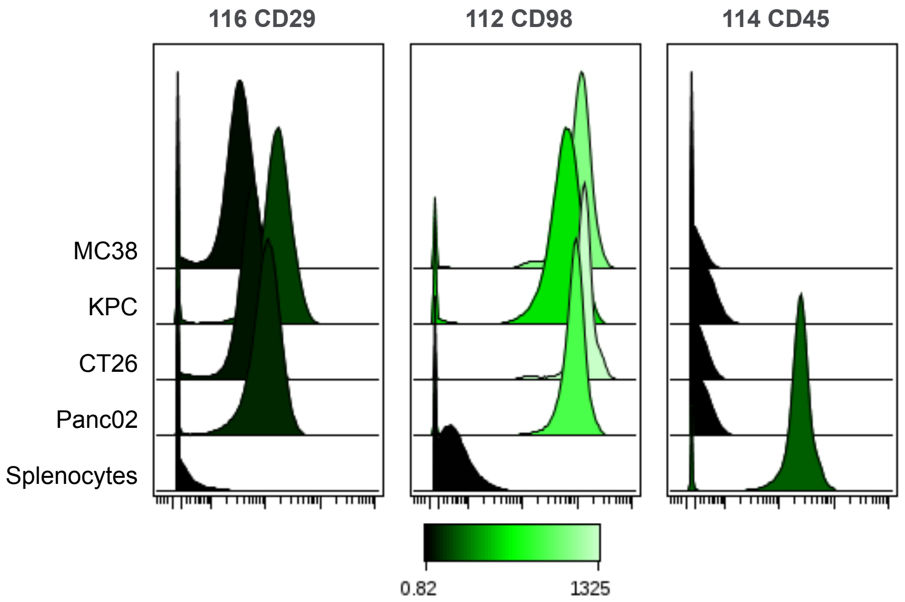

CyTOF was utilized to test the expression of CD29 and CD98 in addition to CD45 which serves as a control. These markers were tested in the following cancer cell lines: MC38, KPC, CT26, and Panc02 in addition to splenocytes to confirm that these antigens are robustly assayable by CyTOF in our cell lines.

Supplementary Figure 3. Testing metal conjugated CD29

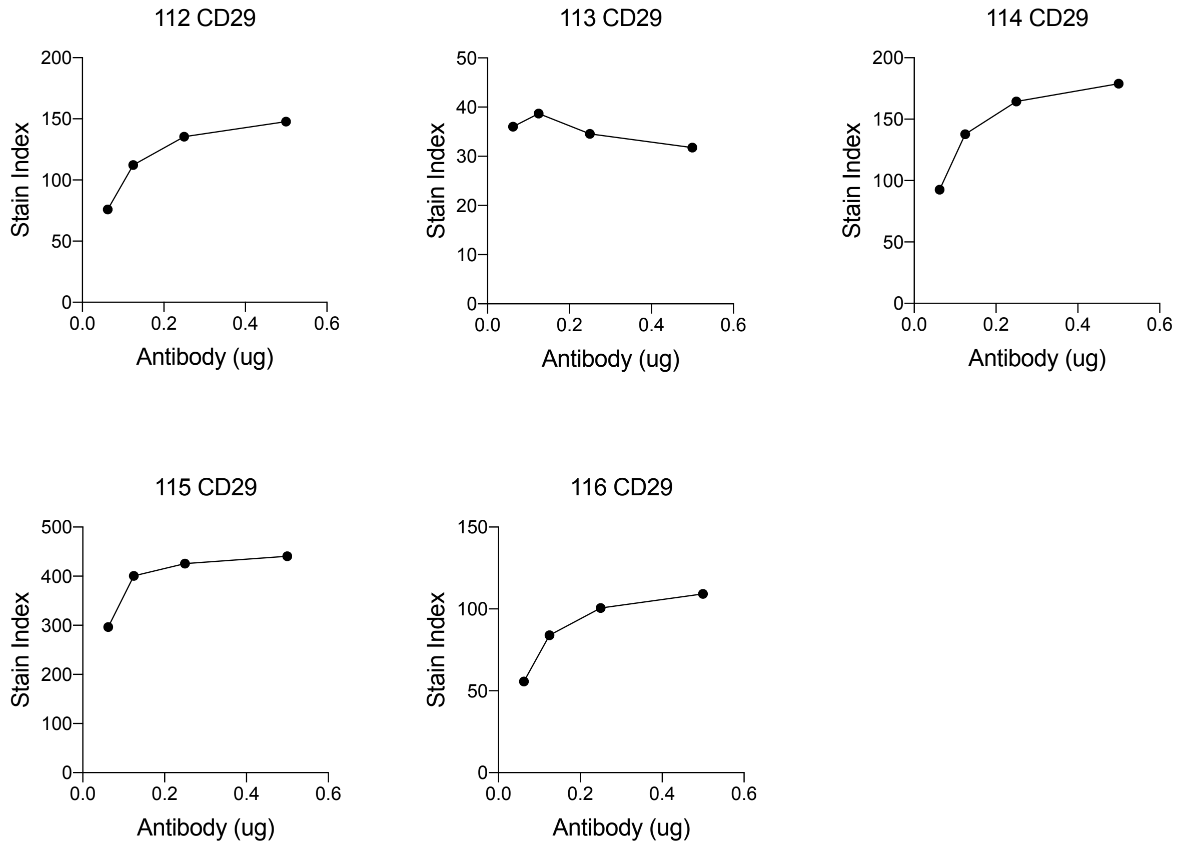

The conjugated anti-CD29 antibodies were tested at four different concentrations (0.0625µg/100µl, 0.125µg/100µl, 0.25µg/100µl, and 0.5µg/100µl) to ensure sufficient staining signal and used to stain MC38. Mean metal intensities of the positive and negative cell populations for each antibody as well as standard deviations of the negative cell populations were used to calculate the staining indexes $(MMI_{pos} - MMI_{neg}/2\sigma_{neg})$ for each antibody at all four concentrations.

Supplementary Figure 4. Hierarchal gating strategy to debarcode 10-plex batch of KPC cells

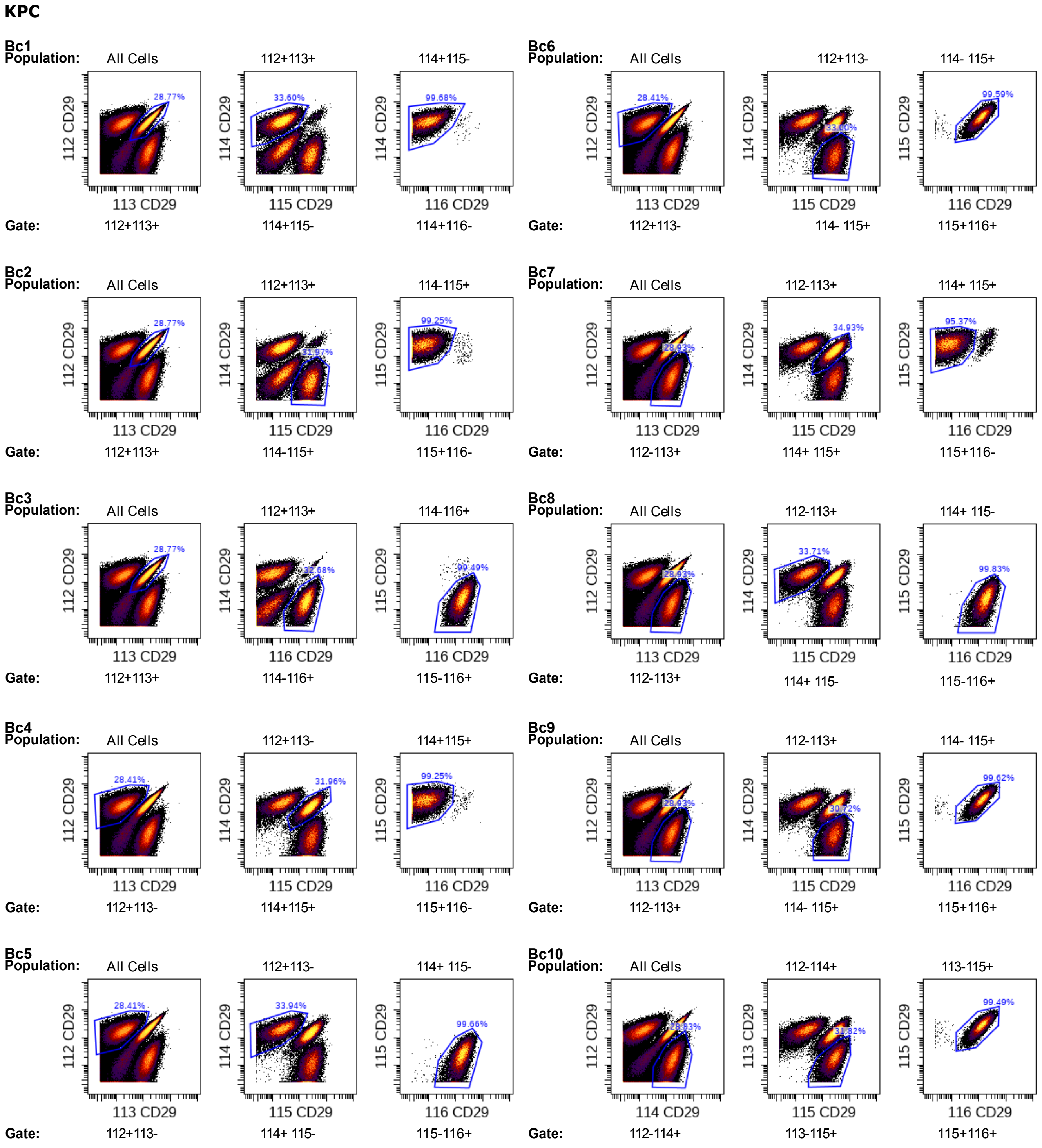

10-plex (5-choose-3) batch of KPC cells are debarcoded by hierarchal gating on Cytobank software by identifying populations that are positive for the three metal channels and negative for the remaining two.

Supplementary Figure 5. Automated debarcoding of 10-plex MC38 and KPC

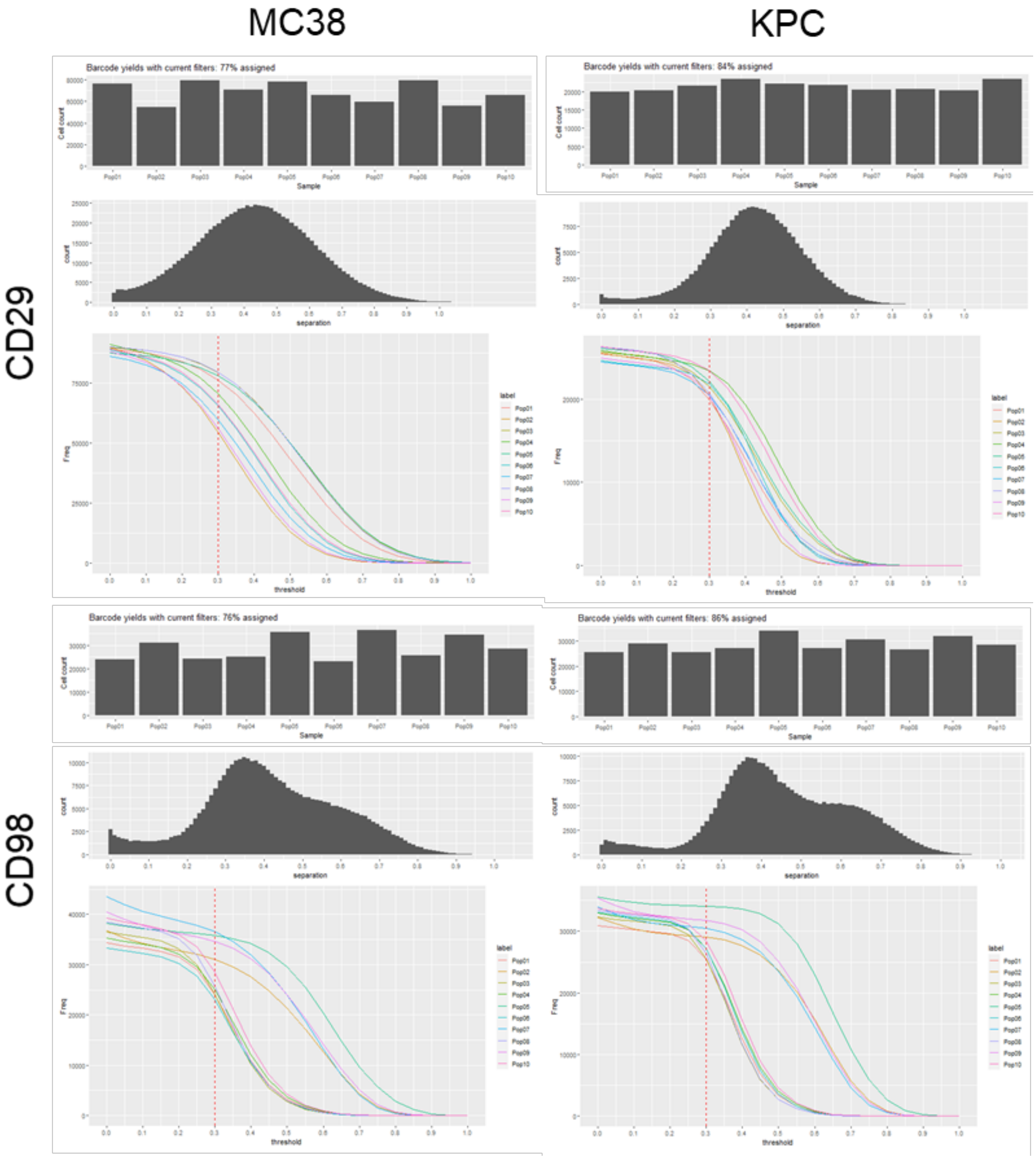

CD29 and CD98-based 10-plex batches of MC38 and KPC cells are debarcoded by Single Cell Debarcoder algorithm (premessa). Bar plots represent the number of cells from each deconvolved sample, retrieving 77%, 84%, 76%, and 86% of the barcoded samples for CD29-based MC38 and KPC batches and CD98-based MC38 and KPC batches, respectively. Separation plot shows barcode yields as a function of the separation threshold (as the threshold increases, the number of cells assigned to each sample decreases; currently selected threshold, 0.3, is displayed as a vertical red line).

Supplementary Figure 6. Comparison of immune cell type abundances between KPC and MC38

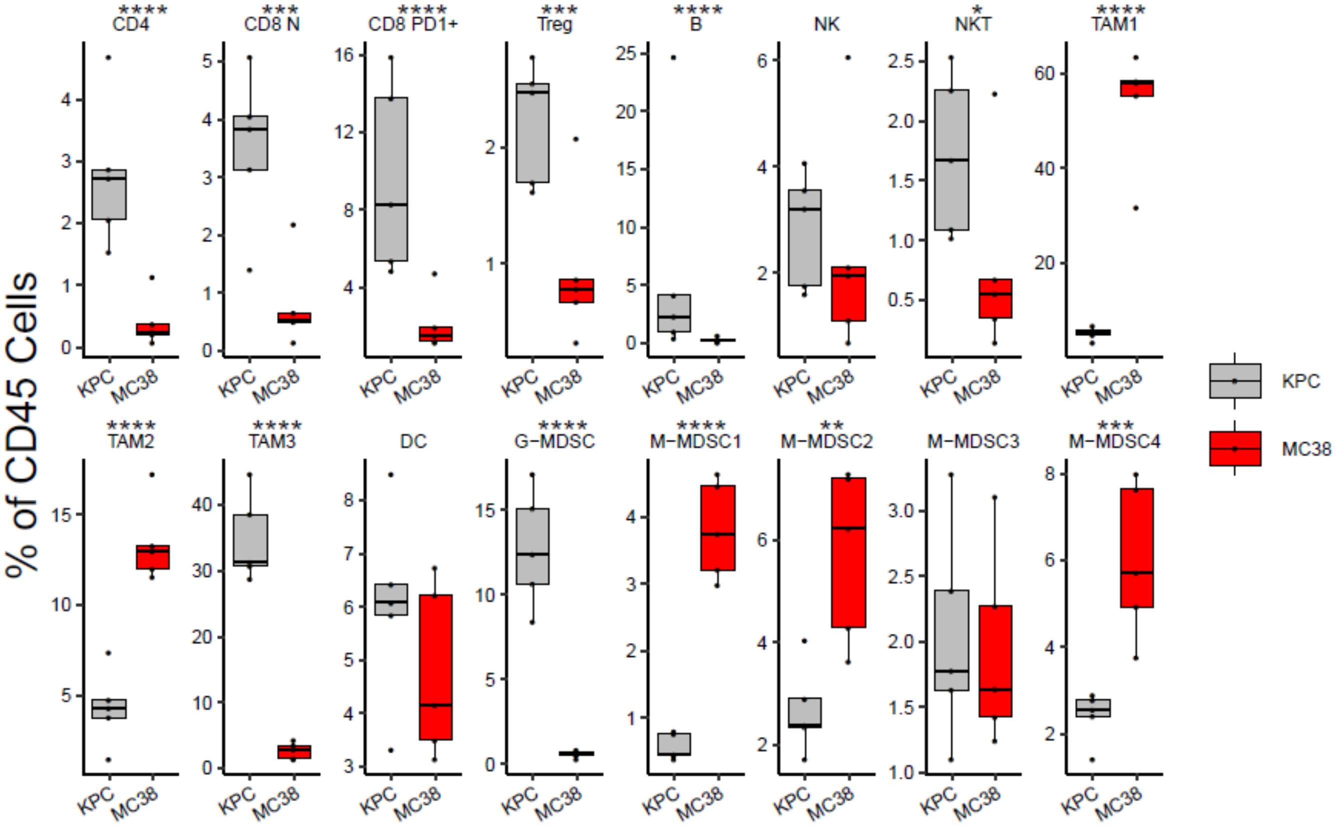

Box plots representation of the same dataset shown on Figure 4D comparing immune cell type abundances within KPC and MC38 tumor models as a percentage of CD45+ cells. FDR adjusted p values by *edgeR* ****<0.0001, ***<0.005, **<0.01, *<0.05. Abbreviations: CD8 N, CD8+ naïve T cells; NK, natural killer cells; NKT, natural killer T cells; TAM, tumor-associated macrophages; DC, dendritic cells; G.MDSC/M.MDSC, granulocytic/ monocytic myeloid-derived suppressor cells.

Supplementary Table S3. Mouse cell line information

Name	Vendor/Source	Origins	Culture Methods
MC38	Kerafast (ENH204-FP)	C57BL6 murine colon adenocarcinoma cells.	Maintained in: • DMEM-based media • 10% FBS • 1% L-glutamine • 1% penicillin/streptomycin • 1% HEPES • 1% sodium pyruvate • 1% non-essential amino acids • 5% CO2 at 37C
CT26.WT	ATCC (CRL-2638)	*N*-nitroso-*N*-methylurethane-induced BALB/c (H-2^d) undifferentiated colon carcinoma(1).	Maintained in: • RPMI 1640 with glutamine 10% FBS • 1% penicillin/streptomycin • 1% sodium pyruvate • 1% non-essential amino acids • 5% CO2 at 37C
B16-F10	ATCC (CRL-6475)	B16 melanoma tumor lines were selected for their ability to form pulmonary tumor nodules(2).	Maintained in: • DMEM-based media • 10% FBS • 1% penicillin/streptomycin • 1% sodium pyruvate • 1% non-essential amino acids • 5% CO2 at 37C
KPC	Johns Hopkins University School of Medicine	Derived from transgenic mice harboring pancreas specific KrasG12D and p53R172H mutations(3).	Maintained in: • RPMI 1640 with glutamine containing 10% FBS • 1% penicillin/streptomycin • 1% sodium pyruvate • 1% non-essential amino acids • 5% CO2 at 37C
Panc02	Johns Hopkins University School of Medicine	Cotton thread-carrying 3-methyl-cholanthrene was implanted into the pancreas of C57BL/6 mice. Of the mice that developed ductal adenocarcinomas, a tumor of C57BL/6 origin (Panc02) was established in a serial subcutaneous transplant(4).	Maintained in: • DMEM-based media • 10% FBS • 1% penicillin/streptomycin • 1% L-glutamine • 10% CO2 at 37C.

The vendor, origin, and culture methods of mouse cell lines used to test antigen expression.

Supplementary Table S4. Mouse immunoprofile panel

Surface Staining

Mass	Marker	Clone	Titration	Source
141	Ly6G	1A8	1:50	Fluidigm
142	CD11c	N418	1:100	Fluidigm
145	CD69	H1.2F3	1:100	Fluidigm
146	F4/80	BM8	1:100	Fluidigm
148	CD11b	M1/70	1:100	Fluidigm
149	CD19	6D5	1:100	Fluidigm
150	Ly6C	HK1.4	1:100	Fluidigm
151	CD25	3C7	1:100	Fluidigm
152	CD3e	145-2C11	1:100	Fluidigm
153	PDL1	10F.9G2	1:100	Fluidigm
155	CD45	30-F11	1:100	Biolegend (c)
159	CD279 (PD-1)	29F.1A12	1:100	Fluidigm
161	CD40	HM40-3	1:50	Fluidigm
164	CD62L	MEL-14	1:100	Fluidigm
168	CD8a	53-6.7	1:50	Fluidigm
170	NK1.1	PK136	1:50	Fluidigm
171	CD44	IM7	1:100	Fluidigm
172	CD4	RM4-5	1:200	Fluidigm
176	B220	RA3-6B2	1:100	Fluidigm
209	MHCII IA/IE	M5/114.15.2	1:100	Fluidigm

Surface mouse immunoprofile panel. Please note, antibody sources are listed, custom antibodies indicated by “(c)” were comprised of purified antibodies from Biolegend conjugated to their respective metal according to the manufacturer’s protocol.

REFERENCES (RELATED TO SUPPLEMENTARY TABLE S3)

1. Wang M et al. Active immunotherapy of cancer with a nonreplicating recombinant fowlpox virus encoding a model tumor-associated antigen. *J. Immunol. Baltim. Md 1950* 1995;154(9):4685–4692.

2. Fidler IJ. Biological behavior of malignant melanoma cells correlated to their survival in vivo. *Cancer Res.* 1975;35(1):218–224.

3. Hingorani SR et al. Trp53R172H and KrasG12D cooperate to promote chromosomal instability and widely metastatic pancreatic ductal adenocarcinoma in mice. *Cancer Cell* 2005;7(5):469–483.

4. Corbett TH et al. Induction and chemotherapeutic response of two transplantable ductal adenocarcinomas of the pancreas in C57BL/6 mice. *Cancer Res.* 1984;44(2):717–726.
